# TRIM24 Expression as an Independent Biomarker for Prognosis and Tumor Recurrence in HNSCC

**DOI:** 10.3390/jpm12060991

**Published:** 2022-06-17

**Authors:** Luise Klapper, Christian Idel, Patrick Kuppler, Tobias Jagomast, Amelie von Bernuth, Karl-Ludwig Bruchhage, Dirk Rades, Anne Offermann, Jutta Kirfel, Sven Perner, Julika Ribbat-Idel

**Affiliations:** 1 Institute of Pathology, University of Luebeck, University Hospital Schleswig-Holstein, Campus Luebeck, 23538 Luebeck, Germany; luise.klapper@student.uni-luebeck.de (L.K.); patrick.kuppler@uksh.de (P.K.); tobias.jagomast@student.uni-luebeck.de (T.J.); amelie.vonbernuth@student.uni-luebeck.de (A.v.B.); anne.offermann@uksh.de (A.O.); jutta.kirfel@uksh.de (J.K.); sven.perner@uksh.de (S.P.); 2 Department of Otorhinolaryngology, University of Luebeck, 23538 Luebeck, Germany; christian.idel@uksh.de (C.I.); karl-ludwig.bruchhage@uksh.de (K.-L.B.); 3 Department of Radiation Oncology, University of Luebeck, University Hospital Schleswig-Holstein, Campus Luebeck, 23538 Lübeck, Germany; dirk.rades@uksh.de; 4 Pathology, Research Center Borstel, Leibniz Lung Center, 23845 Borstel, Germany

**Keywords:** HNSCC, TRIM24, cytoplasmatic, immunohistochemistry, prognostic, local recurrence, progression-free survival, overall survival

## Abstract

Background: Head and neck squamous cell carcinomas (HNSCCs) are among the most common cancers in humans worldwide and have a rather poor prognosis. TRIM24 has various intracellular functions and was identified in other cancer entities as a poor prognostic factor for patients. Methods: The expression of TRIM24 was evaluated by using immunohistochemistry. We used a large and representative cohort of 341 HNSCC patients. Data derived from immunohistochemistry evaluation was correlated with clinicopathological data from HNSCC patients. Results: The TRIM24 expression in HNSCC primary tumors is negatively correlated with the p16 status of the tumor tissues. Primary tumors of patients who developed a local recurrence were significantly more often positive for TRIM24. Kaplan–Meier analyses and Cox regression showed that patients with TRIM24 expressing tumors have significantly worse overall survival and progression-free survival and that TRIM24 expression is independent of other established risk factors. Conclusions: TRIM24 might be a new prognostic biomarker for the survival prognosis and early detection of local recurrences in HNSCC patients. It could be used for risk stratification of HNSCC patients and to identify those patients who are more prone to develop a local recurrence and therefore could profit from more frequent follow-up examinations.

## 1. Introduction

Head and neck squamous cell carcinomas (HNSCCs) are a frequently occurring cancer worldwide [1,2], accounting for 4% of new cancer diagnoses in men in the United States [3]. In Germany, it represents 5% and 2% of new cancer diagnoses in men and women, respectively [4]. It originates from various sites in the head and neck area, including the larynx, oropharynx, hypopharynx, and oral cavity which indicates heterogeneous tumor biology in the group of HNSCCs [5]. Well-known risk factors for the development of HNSCC are alcohol and nicotine consumption [6,7,8]. With the uprising of human papillomavirus (HPV) infections, the prevalence of HPV positive oropharyngeal squamous cell carcinoma (OSCC) is ascending, leaving HPV infection as a new risk factor for HNSCCs [5,9,10,11].

Despite adequate treatment with surgery, chemotherapy, and radiotherapy, the prognosis for patients diagnosed with HNSCC is poor. The overall survival (OS) rates for HNSCC patients have not improved much in the last 10 years [12,13,14]. For instance, the two-year survival rate is 30% and recurrences occur in 30–50% of patients with stage III and IV tumors at the initial diagnosis. Furthermore, patients often suffer from severe long-term treatment related side effects [2,11,15,16,17]. The standard treatment is often a combination of surgery with radiotherapy and/or chemotherapy. After surgery, there are functional and visible impairments for patients, such as visible scars or deformations, voice changes, dysphagia, or chronic pain. Chemotherapy and radiotherapy treatment can lead to xerostomia, as well as to necrosis and fibrosis of the bones and soft tissue in the head and neck area [18]. New treatment options include immune therapies with programmed cell death protein 1 (PD-1) blocking antibodies for recurrent HNSCCs. However, the prognosis for HNSCC patients was not improved to the same extent, as in other cancer entities, such as malignant melanoma [19,20,21]. To improve patient management and survival as well as to reduce the risk of developing LRs (local recurrences), it is important to identify prognostic markers, such as immunohistochemical biomarkers. Ideally, they can help to identify patients with a high risk of a worse OS or development of LRs and introduce them to a more frequent follow-up schedule.

Tripartite Motif-Containing 24 (TRIM24) is a nuclear transcription factor that belongs to the family of tripartite-motif (TRIM) proteins. It has various intracellular functions. TRIM24 works via its function as an E3-ubiquitin ligase as a negative regulator of p53 in the cells and therefore takes influence on the apoptosis [22,23,24]. It also helps to regulate the expression of various receptors such as estrogen receptors (ERs) and retinoic acid-receptor-α (RAR-α) [23]. The nuclear expression or overexpression of TRIM24 is frequently linked to a worse prognosis for patients, e.g., in breast cancer [25], cervical cancer [26], or non-small cell lung cancer (NSCLC) [27]. TRIM24 also seems to play a role for HNSCC patients. It influences the glucose metabolism in HNSCC cells by inducing the expression of glucose transporter GLUT3 and upregulating ATP production and sensitizing the tumor cells to glucose deprivation [28]. Furthermore, TRIM24 expression in HNSCC is linked to increased proliferation and reduced apoptosis rate associated with an overall worse prognosis for the patients [29]. Next to the nuclear TRIM24 expression, the cytoplasmatic TRIM24 expression has also been described in some tumor entities [30,31]. In breast cancer, it also could be correlated with a poorer OS for patients [31]. Nevertheless, to this point, there is only small knowledge about the clinical relevance of cytoplasmatic TRIM24 expression. In this study, we aim to investigate if the cytoplasmatic expression of TRIM24 may serve as a prognostic factor for HNSCC patients.

## 2. Materials and Methods

### 2.1. Patient Data and Tumor Material

The protocol of our study was approved by the Ethics Committee of the University of Luebeck (project code AZ 16-277, date of approval 18 November 2016). The study was conducted in accordance with the Declaration of Helsinki.

We used our large and well-characterized cohort of 341 HNSCC patients for the analysis of TRIM24 expression. The patients were diagnosed in the years 2012–2015 in the Institute for Pathology and treated in the Department for Otorhinolaryngology at the University Hospital Schleswig-Holstein in Luebeck. The clinical data of the HNSCC patients were obtained from clinical and pathological records and afterward anonymized. TNM stages were assessed by the 8th edition of the TNM classification for HNSCC. The five-year OS was defined as the time from diagnosis to last follow-up or death, regardless of the cause of death (all-cause mortality).

Tissue microarrays (TMAs) were assembled from formalin-fixed paraffin-embedded (FFPE) tumor tissues of primary tumors (PTs), lymph node metastases (LNs), distant metastases (DMs), and local recurrences (LRs) as previously described [32,33]. Up to 54 tumor samples and 6 samples of benign tissue from the head and neck region were grouped as triplet cores into one TMA. Each core had a diameter of 1 mm. Three core samples for each tumor were analyzed for a statistical evaluation by calculating the mean value of the three cores.

### 2.2. Immunohistochemistry and Evaluation

Immunohistochemical staining was performed on 4 µm thin sections of FFPE (formalin-fixed, paraffin-embedded) tissue after deparaffinization and antigen retrieval, as previously described [34]. We used the rabbit polyclonal anti-TRIM24 antibody 14208-1-AP (Proteintech Group, Rosemont, IL, USA) with a dilution of 1:75. Staining was performed on a Ventana BenchMark automated staining system (Roche, Basel, Switzerland) with the iView™ DAB Detection Kit (Ventana Medical System, Tuscon, AZ, USA), as previously described [35]. The p16 status was determined by using the mouse monoclonal anti-p16 antibody, clone E6H4™ (p16 CINtec ready to use kit, Roche Ventana Medical Systems, Tucson, AZ, USA).

The stained slides were digitalized and scanned using the Ventana iScan HT scanner (Ventana, Tuscon, AZ, USA) with a 40-fold objective. The software QuPath v. 0.2.2 (Edinburgh, Scotland) was used for viewing the digitalized files [36]. The stained tissue cores were then assessed for a positive or negative TRIM24 staining of tumor tissue. Tumor tissues showed an intratumoral homogeneity in TRIM24 staining, meaning either almost all tumor cells were showing a TRIM24 expression or were not showing any TRIM24 expression. TRIM24 positivity was determined by a visible TRIM24 expression in the tumor cells. Intertumoral heterogeneity of TRIM24 expression patterns within the tumor cells was classified by the localization of the intracellular staining. We defined the following categories: “negative expression” (no detectable TRIM24 expression within the tumor cells), “nuclear expression” (expression of TRIM24 exclusively in the tumor nuclei), “cytoplasmatic expression” (expression of TRIM24 exclusively in the cytoplasm), and “combined expression” (expression of TRIM24 in the tumor nuclei and the tumor cytoplasm). By concentrating on the cytoplasmatic expression of TRIM24, the categories “cytoplasmic expression” and “combined expression” were combined for the statistical analyses as one category of TRIM24 positive tumors. The results were checked by a board-certified pathologist. Samples were distinguished by staining intensity into groups of low, medium, and strong antigen presence as described for other histological markers. Due to the homogenous staining within the samples, the percentage of positively stained cells was neglected for slide assessment [37,38,39].

The evaluation of the immune cell infiltration was performed on hematoxylin and eosin (H & E) stained tumor tissue and was performed by a board-certified pathologist. For details, please refer to Ribbat-Idel et al., 2021 [40].

All steps of the digital analysis were performed on the same computer (Windows 10 based, Microsoft Corporation, Redmond, WA, USA; 15.6″ monitor, resolution 1920 × 1080 px.).

### 2.3. Statistical Analysis and Visualization

IBM^®^ SPSS^®^ Statistics for Windows was used to perform statistical analyses (SPSS Statistics, v. 24, IBM Corp., Armonk, NY, USA). We applied Chi’s square tests to compare TRIM24 expression in different tissue types (PT, LN, LR, and DM, respectively), different tumor sites (hypopharynx, oropharynx, larynx, oral cavity), and various clinicopathological features. Kaplan–Meier analyses and log–rank testing were applied to calculate 60 months OS and progression-free survival (PFS) and to test them for statistical significance. *p*-values below 0.05 were considered to be statistically significant.

We used Microsoft PowerPoint (Microsoft Office 365 ProPlus, Microsoft, Redmont, Washington, DC, USA) to edit the graphics and pictures. All statistical analyses and visualization work were performed on the same computer (Dell Inspiron 5570, Intel-Core i7-8550U, 1.8 GHz, Dell Technologies Inc., Round Rock, TX, USA).

## 3. Results

**Figure 1:**
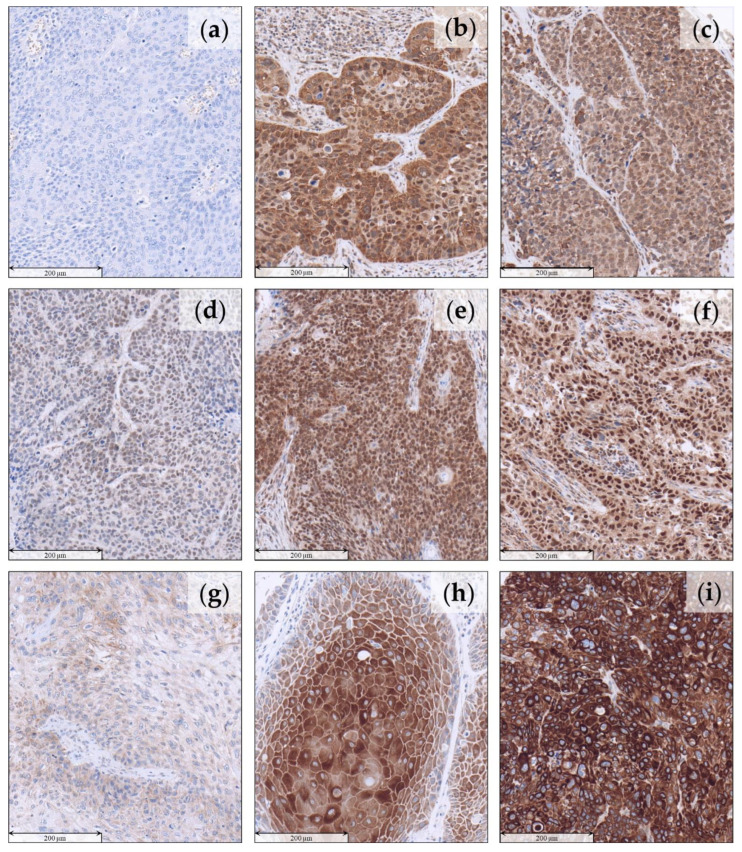
TRIM24 expression patterns in HNSCC tissue. (**a**) “Negative” HNSCC—no detectable TRIM24 expression within the tumor cells. (**b**,**c**) “Combined expression”—expression of TRIM24 in the tumor nuclei and in the tumor cytoplasm. (**d**–**f**) “Nuclear expression”—TRIM24 expression exclusively in the tumor nuclei. (**d**) Low nuclear expression. (**e**) Medium nuclear expression. (**f**) High nuclear expression. (**g**–**i**) “Cytoplasmatic expression”—TRIM24 expression exclusively in the cytoplasm. (**g**) Low cytoplasmatic expression. (**h**) Medium cytoplasmatic expression. (**i**) High cytoplasmatic expression.

For the evaluation of TRIM24 expression in HNSCC, we used our large and well-characterized cohort of HNSCC patients (Supplementary Table S1). Despite TRIM24 being described to be found mainly in the nuclei of tumor cells, we could show a broad variety of expression patterns of TRIM24 in HNSCC tumor cells. We found TRIM24 to be expressed not only in the nuclei of tumor cells but also in the cytoplasm. We classified the different expression patterns into four categories. No detectable TRIM24 expression was labeled as “Negative” (Figure 1a), expression of TRIM24 in the tumor nuclei and the tumor cytoplasm as “Combined expression” (Figure 1b,c), TRIM24 expression exclusively in the tumor nuclei as “Nuclear expression” (Figure 1d–f), and TRIM24 expression exclusively in the cytoplasm as “Cytoplasmatic expression” (Figure 1g–i). In a simple comparison of these four groups, we could see only a difference in the OS when TRIM24 was expressed in the cytoplasm (Supplementary Figure S1). We then divided the “Nuclear expression” and the “Cytoplasmatic Expression” groups into the categories “low” (Figure 1d,g), “medium” (Figure 1e,h), and “high” (Figure 1f,g) expression, depending on the TRIM24 staining intensity. Since only the TRIM24 expression in the cytoplasm had an impact on the OS of HNSCC patients and has not been described in HNSCC yet, we decided to focus on the cytoplasmatic TRIM24 expression in our evaluation. Therefore, we combined the categories “Cytoplasmatic expression” and “Combined expression” and defined them as TRIM24 positive (TRIM24+). TRIM24+ were compared with the remaining tumors, which did not express TRIM24 in their cytoplasm (“Nuclear expression” and “Negative” classified tumors, TRIM24−).

### 3.1. TRIM24 Expression Is Different in Primary Tumors, Lymph Node Metastases, Local Recurrences, and Distant Metastases

**Figure 2:**
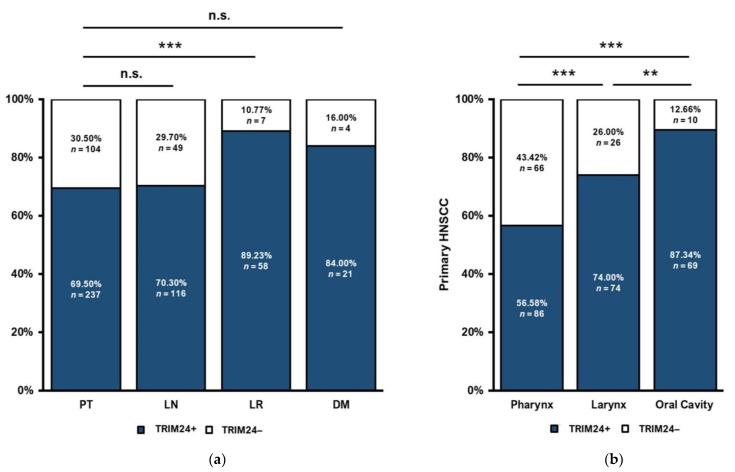
TRIM24 expression in the tissue of PTs, LNs, LRs, and DMs and in different HNSCC tumor sites. (**a**) TRIM24 is expressed more frequently in the tissue of LRs. There are significantly more TRIM24+ LRs than PTs (Chi-square test, *p* = 0.01). There are no significant differences between PTs and LNs and PTs and DMs (Chi-square test, *p* > 0.05). (**b**) Most TRIM24+ tumors could be found in the oral cavity, compared to laryngeal HNSCCs (Chi-square test, *p* = 0.002) and pharyngeal HNSCCs (Chi-square test, *p* < 0.001). Laryngeal HNSCCs are significantly more often TRIM24+ than pharyngeal HNSCCs (Chi-square test, *p* = 0.001). (n.s. = not significant, ** *p* ≤ 0.01, *** *p* ≤ 0.001).

Our cohort contains tissue of HNSCC PTs, LNs, LRs, and DMs. In a first step, we analyzed the differences of the cytoplasmatic TRIM24 expression in the different tumor tissues. The TRIM24 expression of PTs (*n* = 341) and LNs (*n* = 165) and PTs and DMs (*n* = 25) did not differ significantly. In LRs (*n* = 65), significantly more cytoplasmatic TRIM24+ tumors were found than in PT tissue (*p* = 0.001) (Figure 2a).

### 3.2. Distinct TRIM24 Expression in HNSCC Tumor Sites

In the following analyses, we focused on the PTs of our HNSCC cohort. We could observe differences in TRIM24 expression between the different sites of origin of HNSCC PTs. Firstly, there was no significant difference between OSCCs and hypopharyngeal HNSCC PTs. Therefore, they were grouped as pharyngeal PTs (*n* = 152). The proportion of TRIM24+ tumors is significantly lower in pharyngeal HNSCC, than in laryngeal HNSCC (*n* = 100, *p* < 0.001) or in tumors of the oral cavity (*n* = 79, *p* = 0.001). Oral Cavity HNSCCs are showing the significantly highest proportion of TRIM24+ tumors compared to laryngeal HNSCC (*p* = 0.002) and pharyngeal HNSCC (*p* < 0.001) (Figure 2b).

### 3.3. TRIM24 Expression Is Lower in HNSCC Primary Tumors with Lymph Node Metastases

**Figure 3:**
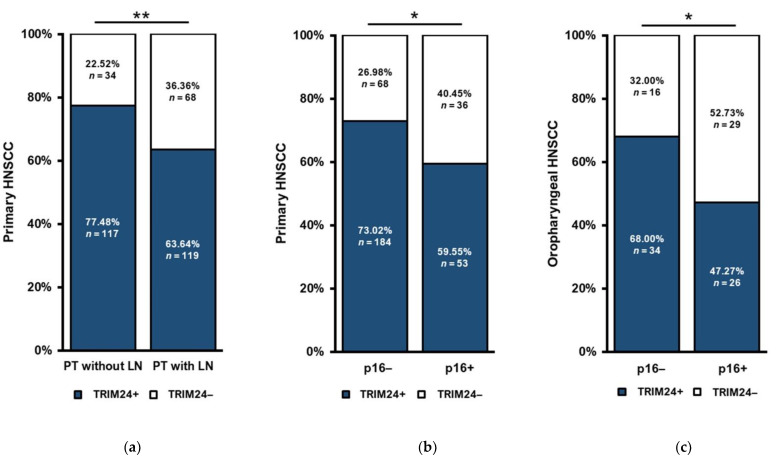
TRIM24 expression in PTs with different N-Stages. (**a**) HNSCC PTs without any LNs at the time of the first diagnosis were significantly more often TRIM24+ than PTs with LNs (Chi-square test, *p* = 0.006). (Chi-square test, *p* > 0.005). (**b**) We compared all HNSCC PTs by their p16 status and TRIM24 expression. P16− PTs are significantly more often TRIM24+ than p16+ PTs (Chi-square test, *p* = 0.018). (**c**) We compared exclusively oropharyngeal HNSCC PTs by their p16 status and TRIM24 expression. P16− PTs were also significantly more often TRIM24+ than p16+ PTs. (n.s. = not significant, * *p* ≤ 0.05, ** *p* ≤ 0.01).

When first diagnosed, 151 patients of our cohort had no known LNs, whereas 187 patients were diagnosed with one or more LNs. We found that the proportion of TRIM24+ PTs was significantly higher in PTs without any LN at the time of initial diagnosis (*p* = 0.006) (Figure 3a).

### 3.4. Association of TRIM24 Expression and the p16 Expression of HNSCC Primary Tumors

We evaluated the expression of p16 as a surrogate marker for HPV infection for the PTs and compared the expression of p16 with the expression of TRIM24. In all PTs (*n* = 341), p16 negative (p16−) PTs showed a significantly more frequent expression of TRIM24 than p16 positive (p16+) PTs (*p* = 0.018) (Figure 3b). We did the same analysis only for OSCC (*n* = 105) in which the p16/HPV status plays a prognostic role and we could show a similar result. P16− PTs showed a higher proportion of TRIM24 positivity than p16+ PTs (*p* = 0.032) (Figure 3c).

### 3.5. TRIM24 Is Expressed More Frequently in HNSCC Primary Tumors with Local Recurrences

**Figure 4:**
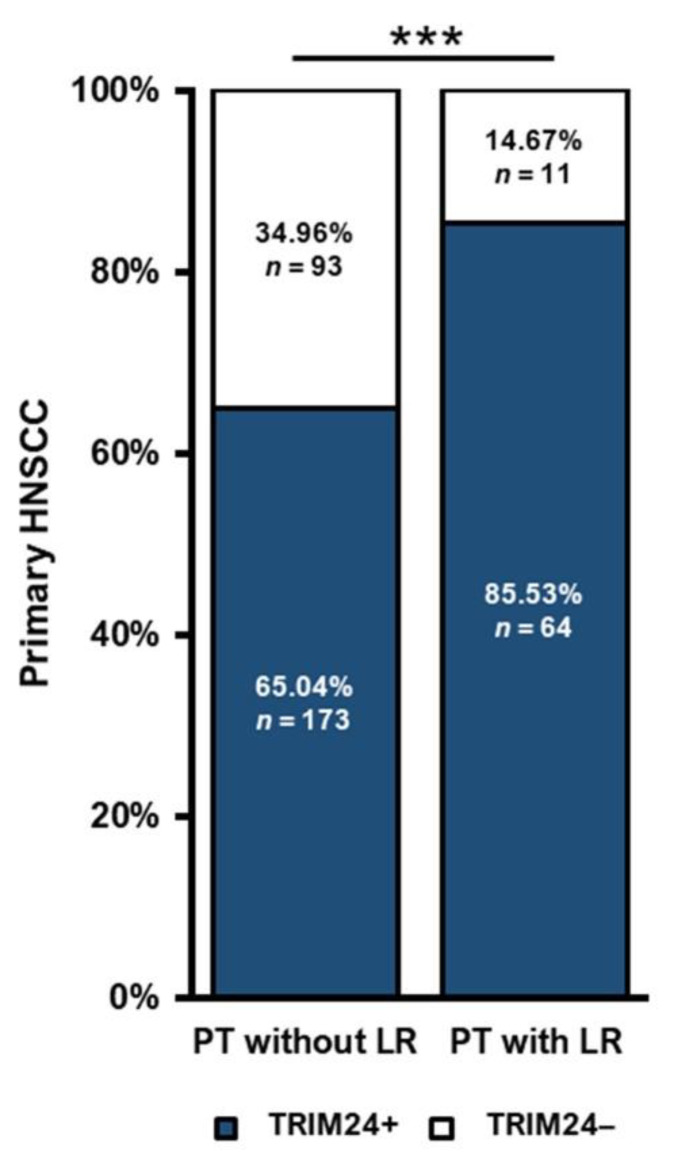
Correlation of TRIM24 expression with the development of a LR and the p16 status in HNSCC. PTs which developed a subsequent LR are significantly more frequent TRIM24+ than PTs without a LR (Chi-square test, *p* = 0.001). (*** *p* ≤ 0.001).

Next, we evaluated if TRIM24 could play a prognostic role for patients who may develop a LR in the course of the disease. To that end, we compared the proportions of TRIM24+ PTs that did or did not develop a LR. Of the 341 PTs in our cohort, 75 PTs developed a subsequent LR, whereas 266 PTs did not develop a LR. A total of 85.53% of PTs that developed a LR were TRIM24+, compared to 65.04% of PTs without a subsequent LR (*p* = 0.001) (Figure 4).

### 3.6. Overall Survival in Patients with TRIM24+ Tumors

**Figure 5:**
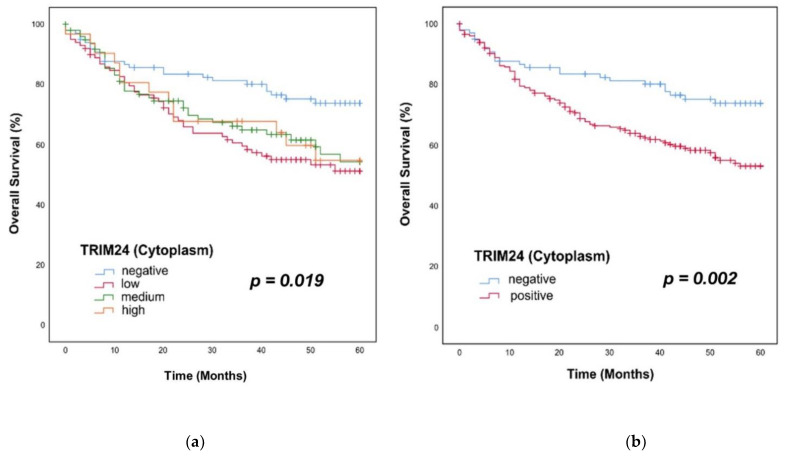
Kaplan–Meier analyses of TRIM24 expressing HNSCC PTs. (**a**) There is a significant difference in the OS rates over 60 months of patients with TRIM24 negative, low, medium, and high expressing PTs (log–rank test, *p* = 0.019). (**b**) We compared all TRIM24 expressing PTs against the TRIM24- tumors. The patients with TRIM24+ PTs show a significantly worse OS over 60 months than patients with TRIM24− PTs (log–rank test, *p* = 0.002).

We applied the Kaplan–Meier method and log–rank testing for survival analyses. For the survival analyses, we divided the group of TRIM24+ PTs into TRIM24 low, medium, and high expressing PTs. We could show a significant difference in the OS of HNSCC patients with TRIM24 negative, low, medium, and high expressing PTs (*p* = 0.019). Low, medium, and high expressing PTs showed similar survival rates within 60 months (Figure 5a). We then compared just TRIM24+ and TRIM24− PTs in the OS, because the subgroups of low, medium, and high TRIM24 staining intensities showed no differences. In TRIM24+ tumors, the OS was significantly worse than in TRIM24− PTs (*p* = 0.002). The survival rate of TRIM24+ patients was 53% after 60 months, versus 74% in TRIM24− patients (Figure 5b). Univariate and multivariate cox regression analyses were applied to identify the TRIM24 expression as an independent prognostic factor for HNSCC patients. In the univariate Cox regression analyses, TRIM24 expression (*p* = 0.003), the p16 expression (*p* = 0.001), the T stage (*p* < 0.001), and the N stage (*p* < 0.001) were identified as significant prognostic factors. In the multivariate Cox regression TRIM24 expression (Hazard Ratio (HR) = 1.89, *p* = 0.007), T stage (HR = 1.906, *p* = 0.001), and N stage (HR = 1.990, *p* < 0.001) could be identified as independent prognostic factors for the OS of HNSCC patients (Table 1).

### 3.7. Progression-Free Survival in Patients with TRIM24+ Tumors

**Figure 6:**
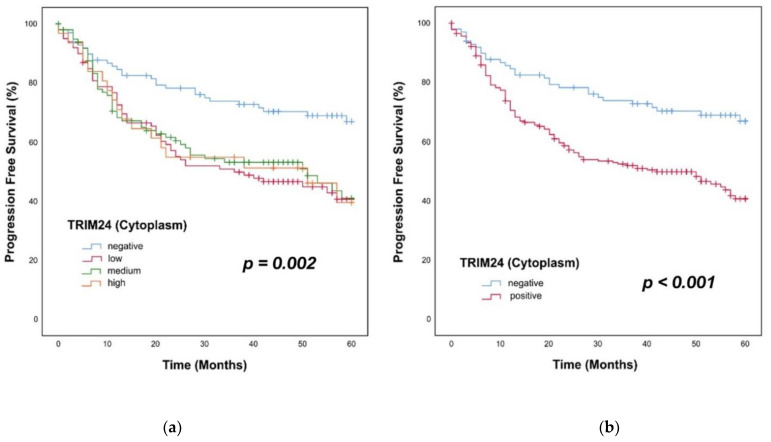
Kaplan–Meier analyses of TRIM24 expressing HNSCC PTs. (**a**) We could show a significant difference in the PFS rates over 60 months of patients with TRIM24 negative, low, medium, and high expressing PTs (log–rank test, *p* = 0.002). (**b**) We compared all TRIM24 expressing PTs against the TRIM24− tumors. The patients with TRIM24+ PTs show a significantly worse PFS over 60 months than patients with TRIM24− PTs (log–rank test, *p* < 0.001).

The same procedure was applied for analyzing the PFS of the patients of our HNSCC cohort. First, we did a Kaplan–Meier analysis and log–rank testing for the comparison of TRIM24 negative, low, medium, and high expressing PTs. Here, we could also show a significantly worse PFS of patients with TRIM24 low, medium, and high expressing PTs against patients with TRIM24− PTs (*p* = 0.002) (Figure 6a). Additionally, when comparing just TRIM24+ and TRIM24− PTs, the patients with TRIM24+ PTs showed a significantly worse PFS than patients with TRIM24− PTs (*p* < 0.001). After 60 months, only 41% of HNSCC patients with TRIM24+ PTs survived without a LR, whereas 69% of patients with TRIM24− PTs could survive without developing a LR (Figure 6b). Again, we applied univariate and multivariate cox regression for the evaluation of the independence of TRIM24 as a prognostic factor. The univariate Cox regression analysis could identify the TRIM24 expression (*p* < 0.001), the p16 expression (*p* < 0.001), the T stage (*p* < 0.001), and the UICC stage (*p* < 0.001) as significant factors for the PFS prognosis of HNSCC patients. In the multivariate Cox regression, the TRIM24 expression (HR = 2.114, *p* < 0.001), the p16 expression (HR = 0.530, *p* = 0.005), the T stage (HR = 1.925, *p* < 0.001), and the N stage (HR = 1.653, *p* = 0.003) could be confirmed as independent prognostic factors for the PFS (Table 2).

### 3.8. TRIM24 Expression Is Independent of T Stage and UICC Stage

**Figure 7:**
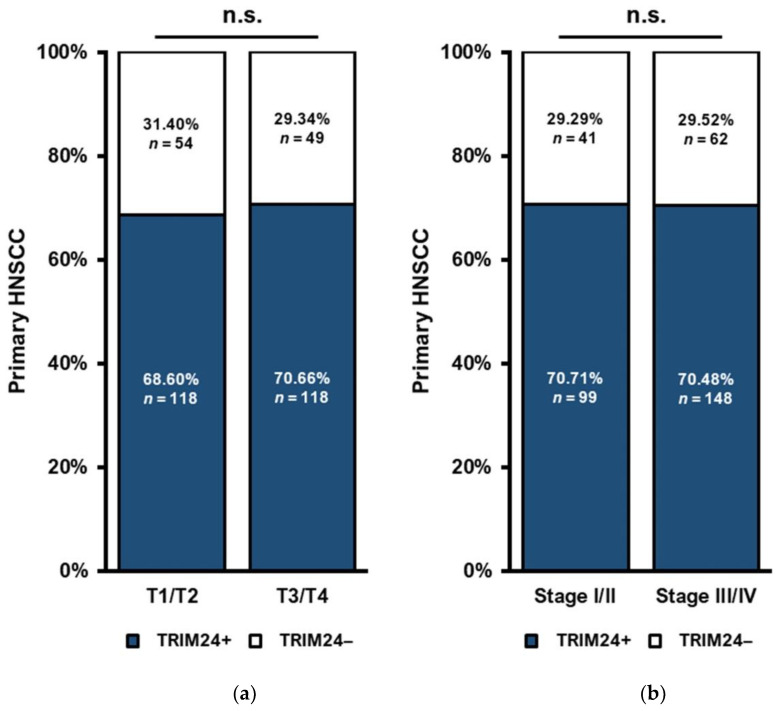
TRIM24 expression in PTs of different T− and UICC-stages. (**a**) There is no significant difference in the proportion of TRIM24 expressing PTs between low T-Stages (T1/2) and higher T-Stages (T3/T4) (*p* > 0.05). (**b**) There is no significant difference in the proportion of TRIM24 expressing PTs between low UICC-Stages (I/II) and higher UICC-Stages (III/IV) (*p* > 0.05). (n.s. = not significant).

We compared the TRIM24 expression in PTs diagnosed at different T stages and UICC stages. No significant differences between the proportion of TRIM24 positive cells between the stages T1 and T2 (*n* = 172) compared to T3 and T4 (*n* = 167) could be found (Figure 7a). Between the UICC stages, I and II (*n* = 130) compared to stages III and IV (*n* = 210), there were no significant differences either (Figure 7b).

There was no association of the TRIM24 expression with age, sex, nicotine, alcohol abuse, or the immune infiltration status of the PTs (Supplementary Table S2).

## 4. Discussion

TRIM24 is involved in many biological processes, including cell differentiation and apoptosis, regulation of gene expression, and DNA repair [41]. TRIM24 overexpression in different cancer entities, including HNSCCs, is predominantly associated with a poor prognosis for cancer patients [10,25,27,29,42,43]. In most studies regarding TRIM24 expression in cancer tissues, the authors focused on the nuclear overexpression of TRIM24 [10,25,27,29,42,44]. However, TRIM24 can not only be found in the nuclei but also in the cytoplasm of tumor cells, e.g., in prostate cancer [30] and breast cancer [31]. This may indicate different functions of TRIM24 in different cancer entities and subcellular compartments. These results are in accordance with our own findings. We found TRIM24 to be expressed in the nuclei of HNSCC tumor cells as well as in the cytoplasm. We could show in our results that only the cytoplasmatic TRIM24 expression has an impact on the OS and PFS of HNSCC patients. Interestingly, the expression levels of TRIM24 (low, medium, and high expression) seem not to have an influence on the prognosis. Our results indicate that cytoplasmatic TRIM24 may play an important role in the tumor biology of HNSCCs. We could show the association of cytoplasmatic TRIM24 with the development of a LR. Furthermore, TRIM24+ tumors were associated with poor OS and PFS in HNSCC patients. This may indicate an important role of the cytoplasmatic TRIM24 in HNSCC e.g., as an E3-ubiquitin ligase. However, the exact molecular mechanisms and biological pathways behind these results are yet to be explored. More functional analyses about the intracellular and cytoplasmatic functions of TRIM24 in cancer cells and especially in HNSCC are necessary.

HNSCCs are a heterogeneous group of squamous cell carcinomas, derived from the epithelial cells of the head and neck region. This includes tumors from the oral cavity, oropharynx, hypopharynx, and larynx. Depending on the site of origin, HNSCCs can show different tumor biology. This can be seen in unequal responses toward radiotherapy, which is worst in hypopharyngeal HNSCCs [45]. Furthermore, the OS of patients with OSCCs can be affected by an infection with the human papillomavirus (HPV), whereas an HPV infection has no impact on the prognosis of tumors in the larynx, hypopharynx, or oral cavity [46]. Our data also indicates differences in the tumor biology of HNSCCs derived at different tumor sites. The most TRIM24 expressing PTs could be found in the oral cavity, with almost 90% TRIM24 positivity. However, the smallest amount of TRIM24+ tumors are derived from the pharynx. Different expression patterns in different tumor sites are an indicator of a different tumor biology. Yet the exact roles of TRIM24 in HNSCCs remain unknown. The topic of diverse tumor biology in HNSCCs needs further investigation to explain differences in therapy response and prognosis in the large group of HNSCC patients.

HPV infection can have an impact on the prognosis of HNSCC patients, especially in patients with oropharyngeal HNSCC. Indeed, HPV positivity generally improves the OS of HNSCC patients [46]. The HPV and the p16 status are established and relevant prognostic factors for HNSCC patients, especially in OSCC [46,47,48]. We investigated if there is a correlation between TRIM24 and the p16 status to state whether TRIM24 is independent of the p16 status. Our results show an inverse relationship between the p16 expression (as a correlate marker for an HPV infection) and the TRIM24 expression. TRIM24+ PTs were more often p16-negative than TRIM24− PTs. This result was shown in oropharyngeal HNSCCs, as well as in all HNSCCs of our cohort. This may indicate a connection in the cellular functions of p16 and TRIM24. While an HPV infection (or the p16 status, respectively) works as a positive prognostic factor for OSCC patients, p16-negative patients have a worse prognosis [46]. This matches our results as p16 negative patients are more often TRIM24+ and therefore have a worse prognosis. We could show in the Cox regression that TRIM24 is independent of other established prognostic factors for HNSCC patients, such as p16 status, T stage, and N stage [47]. Additionally, we found no differences in the TRIM24 expression between PTs of lower T stages (T1/T2) or UICC Stages (UICC I/UICC II) and higher T stages (T3/T4) or UICC stages (UICC III/UICC IV). The UICC stage may be a more relevant prognostic tool as it provides comparability between different HNSCC tumor sites. Therefore, TRIM24 is independent of clinical risk factors and not influenced by the stage of the tumor.

Although p16 (or HPV infection, respectively) works as a positive prognostic marker for HNSCC, the p16 expression in HNSCC could be connected to an advanced nodal stage in PTs [48,49]. This could explain the inverse correlation between TRIM24 expression and the presence of LNs in HNSCC, which we could see during our analyses. Despite TRIM24 being a negative prognostic biomarker for the OS and PFS of patients, TRIM24+ PTs tend to have fewer LNs than TRIM24− PTs. If this effect could only be explained by the association of TRIM24 and p16 expression, or if there are different molecular pathways, which lead to fewer LNs in TRIM24+ PTs remains unclear and needs further investigation. Besides that, an increased TRIM24 expression was positively correlated with a higher nodal stage in other cancer entities [26,42].

Furthermore, we could show that patients with TRIM24+ PTs are associated with the development of a LR. Similar results could be shown in other tumor entities, e.g., prostate cancer [26] and cervical cancer [42]. At this point, we cannot say if TRIM24+ patients may need an improved or intensified treatment strategy because we do not know if TRIM24 has any effect on the sensitivity to radiotherapy or chemotherapy in HNSCC patients. To answer this question, prospective studies and functional analyses of TRIM24 are needed, especially in HNSCCs. An even more important role of TRIM24 could be shown as a new prognostic biomarker. For the clinical management of HNSCC patients, there is still a lack of prognostic factors that may indicate, for example, the development of local recurrences. On the one hand, we can observe patients in the daily clinical work with small PTs in low tumor stages who develop an early LR. On the other hand, there are patients with higher staged PTs who do not develop a LR over years. Therefore, TRIM24 may serve for better risk stratification of HNSCCs. TRIM24+ patients may profit from more frequent follow-up examinations after the primary treatment. This could help to detect and treat LRs earlier in patients who are at risk for LR development. Although most LRs occur within 18 months after the initial treatment [2,15,16,17], we observed the occurrence of LRs at a later point in time as well. So, an improved follow-up schedule should be employed not only for 18 months but for a longer time after the primary treatment if the tumor is TRIM24+. Nevertheless, the clinical benefits of tighter follow-up schedules for high-risk HNSCC patients need to be evaluated by prospective clinical studies. More frequent follow-up schedules might have benefits for the prognosis of HNSCC patients. Nevertheless, they are also associated with higher costs, higher risks, side effects, and more physical and psychological stress for cancer patients, who would have to undergo partly invasive diagnostical procedures more frequently. Additionally, the prognostic value of TRIM24 will have to be validated by further clinical prospective and retrospective studies.

## 5. Conclusions

Cytoplasmatic TRIM24 expression was associated with the occurrence of LRs as well as a worse OS in HNSCC patients.

TRIM24 might serve as a new prognostic biomarker for the risk-stratification of HNSCC patients. Patients with TRIM24 positive tumors may profit from more frequent follow-up schedules. However, the molecular mechanisms and cytoplasmatic functions of TRIM24 in HNSCCs and the prognostic value of TRIM24 will have to be validated in further studies.

## Figures and Tables

**Table 1:** Univariate and multivariate cox regression for 60-months OS.

Variable	Univariate Cox Regression			Multivariate Cox Regression		
	Hazard Ratio	95% Confidence Interval	*p*-Value	Hazard Ratio	95% Confidence Interval	*p*-Value
Cytoplasmatic TRIM24 Expression	1.973	1.260–3.091	0.003 *	1.890	1.194–2.990	0.007 *
P16 Expression	0.478	0.310–0.737	0.001 *	0.641	0.395–1.041	0.072
Grading	1.316	0.909–1.906	0.146			
T Stage (T1 + T2/T3 + T4)	2.289	1.613–3.248	<0.001 *	1.906	1.302–2.791	0.001 *
N Stage (N0 + N1/N2 + N3)	2.252	1.799–3.546	<0.001 *	1.990	1.374–2.882	<0.001 *

* = significant result.

**Table 2:** Univariate and multivariate cox regression for 60-months PFS.

Variable	Univariate Cox Regression			Multivariate Cox Regression		
	Hazard Ratio	95% Confidence Interval	*p*-Value	Hazard Ratio	95% Confidence Interval	*p*-Value
Cytoplasmatic TRIM24 Expression	2.122	1.420–3.170	<0.001 *	2.114	1.396–3.202	<0.001 *
P16 Expression	0.456	0.313–0.666	<0.001 *	0.530	0.338–0.829	0.005 *
Grading	1.040	0.740–1.461	0.823			
T Stage (T1 + T2/T3 + T4)	2.157	1.593–2.920	<0.001 *	1.925	1.373–2.698	<0.001 *
N Stage (N0 + N1/N2 + N3)	1.959	1.457–2.633	<0.001 *	1.653	1.188–2.300	0.003 *

* = significant result.

## Data Availability

The data presented in this study are available on request from the corresponding author.

## Supplementary Materials

The following supporting information can be downloaded at: https://www.mdpi.com/article/10.3390/jpm12060991/s1, Figure S1: Kaplan Meier analyses of TRIM24 expressing HNSCC PTs. There is a significant difference in the OS rates over 60 months of patients with Negative, Cytoplasmatic, Nuclear and Combined TRIM24 expression (log-rank test, *p* = 0.014). Negative and Nuclear TRIM24 expression showed similar survival rates. So did Cytoplasmatic and Combined TRIM24 expression. Table S1: Correlation of TRIM24 expression with clinicopathological features of HNSCC patients. Table S2: Correlation of TRIM24 expression with clinicopathological features of HNSCC patients (Chi’s square test).

## Abbreviations

DM	Distant metastasis
ER	Estrogen receptor
FFPE	Formalin-fixed paraffin-embedded
HE	hematoxylin-eosin
HNSCC	Head and neck squamous cell carcinoma
HPV	Human papillomavirus
LN	Lymph node metastasis
LR	Local recurrence
NSCLC	non-small cell lung cancer
OS	Overall survival
OSCC	Oropharyngeal squamous cell carcinoma
p16−	p16 negative tumors
p16+	p16 positive tumors
PD-1	programmed cell death protein 1
PFS	progression-free survival
PT	Primary tumor
RAR-α	Retinoic acid-receptor-α
TMA	Tissue microarray
TRIM	Tripartite Motif
TRIM24−	TRIM24 negative tumors
TRIM24	Tripartite Motif-Containing 24
TRIM24+	TRIM24 positive tumors
