# Supplementary material for: TRIM24 Expression as an Independent Biomarker for Prognosis and Tumor Recurrence in HNSCC
Source: J Pers Med. 2022 Jun 17;12(6):991. doi: 10.3390/jpm12060991 (PMC9225579; doi:10.3390/jpm12060991)
Supplement: Supplementary file 1 [file jpm-12-00991-s001.zip › jpm-1732989-supplementary.pdf]

Journal of
Personalized Medicine

Supplementary Materials

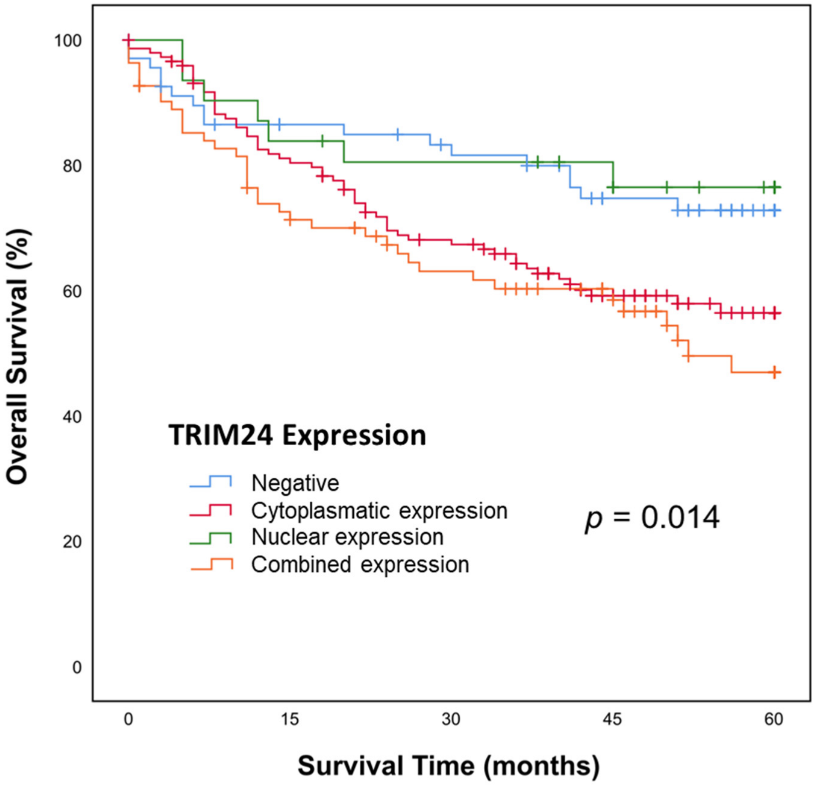

Figure S1. Kaplan Meier analyses of TRIM24 expressing HNSCC PTs. There is a significant difference in the OS rates over 60 months of patients with Negative, Cytoplasmatic, Nuclear and Combined TRIM24 expression (log-rank test, p = 0.014). Negative and Nuclear TRIM24 expression showed similar survival rates. So did Cytoplasmatic and Combined TRIM24 expression.

Table S1. Clinicopathological characteristics and cytoplasmatic TRIM24 expression in HNSCC.

Variable		Cytoplasmatic TRIM24 Expression		Total
		n Positive	*n* Negative	
Tissue Type	Primary Tumor	237	104	341
	Lymph Node Metastases	116	49	165
	Local Recurrences	58	7	65
	Distant Metastases	21	4	25
Tumor Site	Pharynx	86	66	152
	Larynx	74	26	100
	Oral Cavity	69	10	79
	n.a.			10
Sex	Female	50	27	77
	Male	186	77	263
	n.a.			1
Age (years)	<61	108	48	156
	>61	127	56	183
	n.a.			2
T stage *	T1	51	21	72
	T2	67	33	100
	T3	70	26	96
	T4	48	23	71
	n.a.			2
N Stage *	N0	117	34	151
	N1	24	29	53
	N2	61	23	84
	N3	34	16	50
	n.a.			3
UICC stage *	I	44	25	69
	II	45	16	61
	III	42	18	60
	IV	106	44	150
	n.a.			1
Lymph Node Metastases	Yes	119	68	187
	No	117	34	151
	n.a.			3
Distant Metastases	Yes	10	4	14
	No	224	99	323
	n.a.			5
Local Recurrence	Yes	64	11	75
	No	173	93	266
	n.a.			0
Immune Infiltration	Hot	47	19	66
	Excluded	99	47	146
	Cold	48	21	69
	n.a.			60
Alcohol Consumption	Yes	105	38	143
	No	123	62	185
	n.a.			13
Nicotine Consumption	Yes	202	84	286
	No	22	14	36
	n.a.			19
P16 Status	Positive	53	36	89
	Negative	184	68	252
	n.a.			0

n.a. = not available, * = assessed after the 8th edition of TNM classification.

Table S2. Correlation of TRIM24 expression with clinicopathological features of HNSCC patients (Chi's square test).

Clinicopathological Features		Number of Patients	Cytoplasmatic TRIM24 Expression		*p*-Value
			% Positive	% Negative	
Age	<61 years	156	69.23%	30.77%	0.973
	>61 years	183	69.40%	30.60%	
Sex	Female	77	64.94%	35.06%	0.332
	Male	263	70.72%	29.28%	
Alcohol Consumption	Yes	143	73.43%	26.57%	0.176
	No	185	66.49%	33.51%	
Nicotine Consumption	Yes	286	70.63%	29.37%	0.242
	No	36	61.11%	38.89%	
Immune Infiltration	"Cold"	69	69.57%	30.43%	0.913
	"Hot" + "Excluded"	212	68.87%	31.13%	

* = significant result.
